# Transcriptome Modifications in Porcine Adipocytes via Toll-Like Receptors Activation

**DOI:** 10.3389/fimmu.2019.01180

**Published:** 2019-05-29

**Authors:** Manami Igata, Md. Aminul Islam, Asuka Tada, Michihiro Takagi, A. K. M. Humayun Kober, Leonardo Albarracin, Hisashi Aso, Wakako Ikeda-Ohtsubo, Kenji Miyazawa, Kazutoyo Yoda, Fang He, Hideki Takahashi, Julio Villena, Haruki Kitazawa

**Affiliations:** ^1^Food and Feed Immunology Group, Laboratory of Animal Products Chemistry, Graduate School of Agricultural Science, Tohoku University, Sendai, Japan; ^2^Livestock Immunology Unit, International Education and Research Centre for Food and Agricultural Immunology (CFAI), Graduate School of Agricultural Science, Tohoku University, Sendai, Japan; ^3^Department of Medicine, Faculty of Veterinary Science, Bangladesh Agricultural University, Mymensingh, Bangladesh; ^4^Department of Dairy and Poultry Science, Chittagong Veterinary and Animal Sciences University, Chittangong, Bangladesh; ^5^Laboratory of Immunobiotechnology, Reference Centre for Lactobacilli (CERELA-CONICET), San Miguel de Tucumán, Argentina; ^6^Scientific Computing Laboratory, Computer Science Department, Faculty of Exact Science and Technology, National University of Tucuman, San Miguel de Tucumán, Argentina; ^7^Cell Biology Laboratory, Graduate School of Agricultural Science, Tohoku University, Sendai, Japan; ^8^Technical Research Laboratory, Takanashi Milk Products Co., Ltd., Yokohama, Japan; ^9^Laboratory of Plant Pathology, Graduate School of Agricultural Science, Tohoku University, Sendai, Japan; ^10^Plant Immunology Unit, International Education and Research Centre for Food and Agricultural Immunology, Graduate School of Agricultural Science, Tohoku University, Sendai, Japan

**Keywords:** transcriptome, adipocytes, microarray, TLRs (Toll-like receptors), immunometabolism, pig

## Abstract

Adipocytes are the most important cell type in adipose tissue playing key roles in immunometabolism. We previously reported that nine members of the Toll-like receptor (TLR) family are expressed in an originally established porcine intramuscular pre-adipocyte (PPI) cell line. However, the ability of TLR ligands to modulate immunometabolic transcriptome modifications in porcine adipocytes has not been elucidated. Herein, we characterized the global transcriptome modifications in porcine intramuscular mature adipocytes (pMA), differentiated from PPI, following stimulation with Pam3csk4, Poly(I:C) or LPS which are ligands for TLR2, TLR3, and TLR4, respectively. Analysis of microarray data identified 530 (218 up, 312 down), 520 (245 up, 275 down), and 525 (239 up, 286 down) differentially expressed genes (DEGs) in pMA following the stimulation with Pam3csk4, Poly(I:C), and LPS, respectively. Gene ontology classification revealed that DEGs are involved in several biological processes including those belonging to immune response and lipid metabolism pathways. Functionally annotated genes were organized into two groups for downstream analysis: immune response related genes (cytokines, chemokines, complement factors, adhesion molecules, and signal transduction), and genes involved with metabolic and endocrine functions (hormones and receptors, growth factors, and lipid biosynthesis). Differential expression analysis revealed that *EGR1, NOTCH1, NOS2, TNFAIP3, TRAF3IP1, INSR, CXCR4, PPARA, MAPK10*, and *C3* are the top 10 commonly altered genes of TLRs induced transcriptional modification of pMA. However, the protein-protein interaction network of DEGs identified *EPOR, C3, STAR, CCL2*, and *SAA2* as the major hub genes, which were also exhibited higher centrality estimates in the Gene-Transcription factor interaction network. Our results provide new insights of transcriptome modifications associated with TLRs activation in porcine adipocytes and identified key regulatory genes that could be used as biomarkers for the evaluation of treatments having immunomodularoty and/or metabolic functional beneficial effects in porcine adipocytes.

## Introduction

The innate immune system recognizes infectious microbial pathogens through germ line-encoded patterns recognition receptors (PRRs), such as Toll-like receptors (TLRs), and nucleotide-binding oligomerization domain (NOD)-like receptors (1). These receptors interact with the evolutionarily conserved microbial structures known as microbial associated molecular patterns (MAMPs), including lipopolysaccharides (LPS), lipoteichoic acids (LTA), peptidoglycan (PGN), and double stranded viral RNA, which are essential for the survival of microorganisms (2). In addition, PRRs also recognize endogenous damage-associated molecular patterns (DAMPs) derived from dead cells or injury (3), such as free fatty acids, cholesterol, high glucose concentration, ceramides, and urate crystals (4). Although low levels of DAMPs are beneficial during tissue repair, excessive amounts induce chronic low-grade inflammation in various tissue including adipose tissues. Deregulated inflammation in the adipose tissue is involved in the development metabolic disorders like obesity, atherosclerosis, and type-2 diabetes mellitus (5). Therefore, elucidation of the cellular transcriptome modifications in adipocytes associated with the activation of their PRRs is of great importance to understand in more depth the physiopathological mechanisms involved in the metabolic diseases with an inflammatory component and to propose alternatives to prevent them.

TLRs are an important family of PRRs with capacity to sense several types of MAMPs and thereby trigger inflammatory responses (6). Depending on the cellular localization TLRs can be categorized into two subgroups: trans-membrane (such as TLR1, TLR2, TLR4, TLR5, TLR6, and TLR11) and intracellular (such as TLR3, TLR7, TLR8, and TLR9) receptors (6, 7). Early in the immune response TLR ligation induces gene transcription leading to inflammation, tissue repair and initiation of adaptive immunity (6, 8, 9). Previous studies reported that Pam3csk4 is recognized at the cell membrane by TLR1/2 as a mimic of bacterial lipopeptides (10). Poly(I:C), a synthetic double-stranded RNA is recognized in the endosome by TLR3 (11), while LPS is recognized by TLR4 sequentially at the cell membrane and endosome (8, 12). Upon binding to respective ligands, TLRs recruit a set of specific adapter molecules such as myeloid differentiation primary response gene 88 (MyD88), Toll/interleukin-1 receptor (TIR) domain-containing adapter protein (TIRAP), TIR-domain-containing adapter-inducing interferon-β (TRIF), or TRIF-related adapter molecule (TRAM) to initiate the downstream signal transductions that lead to the activation of different transcriptional factor such as nuclear factor-kappa B (NF-kB), activator protein-1 (AP-1), and interferon regulatory factor (IRF) (2, 6). The transcription factors specifically signal the cells to secrete proinflammatory cytokines and chemokines, type-I interferon, and antimicrobial peptides (6), which coordinately induce inflammatory responses.

The adipose tissue is a highly active organ capable of integrating metabolic, endocrine, and immune functions into a single entity that plays a crucial part on systemic homeostasis (4, 13). In addition to its role as an endocrine gland with pleotropic function in the metabolism (14), adipose tissue is increasingly becoming recognized as part of the innate immune system (15, 16). Adipose tissue contains several distinct groups of cells including mature adipocytes, pre-adipocytes, fibroblasts, M1/M2 macrophages; neutrophils, dendritic cells, eosinophils, and endothelial cells (17). Pre-adipocytes have the ability to differentiate into mature adipocyte according to the energy balance (17, 18). Morphologically, adipocytes are spherical cells with a single large lipid droplet formed by triglycerides that account >90% of the cell's volume (19). Mature adipocytes are functionally the most important cell type in adipose tissue and play roles in storing triglycerides and systemic energy balance (20), as well as in antigen presentation (21). Upon stimulation by MAMPs and/or DAMPs, mature adipocytes secret a wide variety of cytokines and other mediators, which are able to contribute to the generation of both local and systemic immune responses (13, 22).

Different transcriptomic analytic approaches have been employed to identify key regulatory genes in response to multi-TLR activation in white blood cells (23) and macrophages (24). Transcriptomic studies aimed to elucidate the gene expression changes after *in vitro* stimulation with different MAMPs have been also performed in human adipocytes (15, 25). However, there is no detailed information on immunotranscriptomic responses following TLRs activation in the porcine adipocytes. Pigs are considered as one of the closest approximate animal models for studying human diseases because of their anatomical, physiological and immunological similarities with humans (26). By using a porcine intramuscular pre-adipocyte (PIP) cell line originally established by our group (27), we recently demonstrated that nine TLRs (TLR 1-9) are expressed in PIP and differentiated porcine mature adipocytes (pMA) (28). Importantly, TLR2 and TLR4 showed significantly higher expression in pMA as compared to PIP while TLR3 showed higher expression in PIP than that of pMA (28). We therefore, aimed herein to characterize the global transcriptome modifications downstream of TLR2, TLR3, and TLR4 activation in pMA. We obtained a global overview of the modulation of the transcriptomic response in pMA and its association with immune, metabolic and endocrine responses. Results of the present study indicated that several immune genes including *EGR1, NOTCH1, NOS2, TNFAIP3, TRAF3IP1, INSR, CXCR4, PPARA, MAPK10*, and *C3* were differentially modulated in pMA cells after TLR ligation. Our results provide new insights of transcriptome modifications associated with TLRs activation in porcine adipocytes and indicate that pMA cells are an interesting tool to study *in vitro* the immune responses triggered by TLR2, TLR3, or TLR4 in this cell population.

## Materials and Methods

### Cell Line, Culture Condition, and Differentiation

Porcine intramuscular pre-adipocyte (PIP) cell line was previously established by our group (27), which was derived from marbling muscle tissue of the musculus longissimus thoracis from female Duroc pig. Culture conditions for inducing adipogenesis were performed according to our previous work (28), In brief, PIP cells (between the 26th and 35th passages) were maintained in Dulbecco's modified Eagle medium (DMEM; Gibco™, Paisley, Scotland, UK) supplemented with 10% (v/v) fetal calf serum (FCS; Sigma-Aldrich, Tokyo, Japan), 100 U/ml penicillin and 100 μg/ml streptomycin (Gibco™ 15140122, Life Technologies) as a growth medium. The PIP cells were plated at density of 2.5 × 10^4^/cm^2^ in 6-well cell culture plates (BD Falcon, Tokyo, Japan), and incubated at 37°C in a humidified atmosphere of 5% CO_2_. The 4-day post-confluent PIP cells were fed with differentiation medium for another 4 days to yield the differentiated adipocyte. The differentiation medium composed with DMEM containing 10% FCS and 50 ng/ml insulin (swine, Sigma-Aldrich, Tokyo, Japan), 0.25 μM dexamethasone (Sigma-Aldrich), 2 mM octanoate (Wako, Osaka, Japan), 200 μM oleate (Ardorich, Milwaukee, WI, USA), 100 U/ml penicillin, and 100 μg/ml streptomycin. The medium was changed every day. The differentiation of PIP into functionally matured porcine mature adipocyte (pMA) was confirmed by detecting the presence of intracellular lipid droplets with Oil red O staining according to our previous publication (28). Briefly, cells were rinsed three times in Dulbecco's Phosphate-Buffered Saline and then fixed in 10% (v/v) formaldehyde for 30 min. Subsequently, the fixed cells were rapidly rinsed with MiliQ water. Finally, 0.5% Oil red O (Sigma-Aldrich, Tokyo, Japan) in isopropanol was added to the cells for 5 min to visualize lipid droplets stained red. The cytosolic triglyceride content was analyzed using LabAssay™ triglyceride kit (FUJIFILM Wako Chemicals USA, Corp.) according to the manufacturer's protocol. In addition, qRT-PCR-based (method followed as described later in this section) expression of specific marker genes were also evaluated for adipocyte maturation.

### Stimulation to Porcine Mature Adipocyte (pMA) by TLR Ligands

Synthetic analogs for three Toll-like receptors: TLR2, TLR4 and TLR3; were used to mimic the inflammatory response induced by gram positive bacteria, gram negative bacteria, and by virus infection, respectively. Pam3csk4, Poly(I:C), and LPS were used as ligands for TLR2, TLR3, and TLR4, respectively. For optimizing the dose of ligands, we first stimulated pMA cells (2.5 × 10^4^/cm^2^) with serial dilutions of each ligand and evaluated CCL2 expression. The optimal doses were selected according to their ability to increase CCL2 expression at least five-folds as compared to that of control (data not shown). The pMA cells were seeded at density of 2.5 × 10^4^/cm^2^ in 6 well or 12 well plates (BD Falcon, Tokyo, Japan). The 4-day post-confluent pMA cells were stimulated either with Pam3csk4 (10 ng/ml), Poly(I:C) (0.1 μg/ml), or LPS (0.1 μg/ml) at 37°C with 5% CO_2_ for 12 h.

### RNA Isolation and Quality Control

Total RNA was isolated from the ligand-treated and control pMA cells using PureLink RNA Mini Kit (Life Technology Inc., USA) along with on-column DNase treatment. RNA integrity, quality and quantity were evaluated with microcapillary electrophoresis (2100 Bioanalyzer, Agilent Technologies, Santa Clara, CA, USA) using the Agilent RNA 6000 Nano kit (Agilent Technologies, Santa Clara, CA, USA). Only samples with RNA integrity number (RIN) of >8 were used for this gene expression study.

### Microarray Hybridization

The microarray hybridization was performed with Porcine Gene Expression Microarray 4 × 44 K oligonucleotide slide (v2.0, Agilent Technologies, Santa Clara, CA, USA) containing 43,803 probes for the identification of known genes of the porcine transcriptome. The microarray experiment was conducted at Hokkaido System Science Co., according to the one-color Microarray-based Gene Expression Analysis protocol v6.7 (Agilent Technologies, Santa Clara, CA, USA). For each samples, 200 ng of total RNA was converted into cDNA by reverse transcription. The cDNA was subsequently transcribed into cRNA and labeled with cyanine 3 (Cy3). About 1.65 μg of labeled cRNA was mixed with hybridization buffer and hybridized on microarray slide (4 samples in each slide) for 17 h at 65°C with constant rotation. After hybridization, microarrays were cleaned with Gene Expression wash buffer and scanned with High-Resolution Microarray Scanner (Agilent Technologies, Santa Clara, CA, USA). The Feature Extraction software (v10.7.3.1, Agilent Technologies, Santa Clara, CA, USA) was used for detailed analysis of scanned images including filtering the outlier spots, background subtraction from features and dye normalization. The spot intensity data for individual sample were extracted for statistical analysis.

### Statistical Analysis of Microarray Data

The normalization and differential expression analysis of microarray data were performed with GeneSpring GX software (v13.1, Agilent Technologies, USA). The log_2_ transformed expression values of probes were normalized based on 75 percentile shifts. In order to determine the TLR-ligand induced differential expression of genes, an unpaired *t*-test was performed between untreated control and TLR-ligand stimulated samples. The pairwise comparisons were performed between control and each of the three TLR-ligand stimulations to detect the differentially expressed genes. Benjamini and Hochberg (B-H) adjustment method was applied for multiple test correction. Significant differentially expressed genes were selected on the basis of two criteria: an adjusted *p*-value (FDR, false discover rate) of <0.05, and a cutoff in fold change of at least 1.5. The human orthologs gene symbols of DEGs were determined using dbOrtho panel of the bioDBnet tool (29) which were used for downstream functional analyses.

### Gene Ontology (GO) and Pathway Analyses

For biological interpretation of differential gene expressions, GO enrichment and pathway analysis was performed using the Database for Annotation, Visualization, and Integrated Discovery (DAVID, v6.8) (30). Human orthologous symbols of DEGs were uploaded to the DAVID web portal and the official gene symbol was chosen as identifier. Then enriched biological themes, particularly GO terms and KEGG pathways were extracted. In the analysis, GO terms and KEGG pathways with an FDR-adjusted *p* < 0.05 were retained.

### Network Enrichment Analyses

In order to visualize TLR ligands-induced transcriptional network as well as to identify the regulatory genes, the sub-network enrichment analysis was performed using NetworkAnalyst online tool (31). This tool uses the InnateDB protein-protein interaction datasets comprised of 14,755 proteins and 145,955 literature-curated interactions for human (32). Human orthologous gene symbols of the common DEGs from all three stimulation were uploaded into the NetworkAnalyst to construct the interaction network based on Walktrap algorithm taking only direct interaction of seed genes. The network was depicted as nodes (circles representing genes) connected by edges (lines representing direct molecular interactions). Two topological measures such as degree (number of connections to the other nodes) and betweenness (number of shortest paths going through the nodes) centrality were taken into account for detecting highly interconnected genes (Hubs) of the network. Nodes having higher degree and betweenness were considered as potentially important hubs in the cellular signal trafficking. In addition, a gene regulatory network focusing the adipose tissue specific gene-transcription factor (Gene-TF) interaction network was also constructed using the NetworkAnalyst tool (31). For constructing the Gene-TF network, transcription factor and gene target data derived from the ENCODE ChIP-seq data were used. Only peak intensity signal <500 and the predicted regulatory potential score <1 were included based on BETA Minus algorithm (33).

### Validation of Microarray Expression by qRT-PCR

Two-step real-time PCR (qRT-PCR) was performed to confirm the microarray results by quantifying expression of selected mRNAs in pMA. Primer sequences are presented in Table 1. Total RNA was isolated from each sample using TRIzol reagent (Invitrogen, Carlsbad, CA, USA) followed by treated with gDNA Wipeout Buffer (Qiagen, Tokyo, Japan). All cDNAs were synthesized using the Quantitect reverse transcription Kit (Qiagen, Tokyo, Japan) according to the manufacturer's recommendations. The qRT-PCR was performed using 7300 real-time PCR system (Applied Biosystems, Warrington, UK) using the TaqMan^®^ gene expression assay kit (Life Technologies) and TaqMan^®^ Universal Master Mix II, with UNG (Applied Biosystems, Warrington, UK). The PCR cycling conditions were 2 min at 50°C, followed by 10 min at 95°C, and then 40 cycles of 15 s at 95°C, 1 min at 60°C. The reaction mixtures contained 2.5 μl of sample cDNA, 1 μl gene expression assay and 10 μl TaqMan^®^ Universal Master mix II, with UNG, and 6.5 μl distilled water. According to the minimum information for publication of quantitative real-time PCR experiments guidelines, Beta actin (ACTB) was used as a house-keeping gene because of its high stability across various porcine tissues (34, 35). Relative index was calculated as the ratio of target mRNA expression to ACTB. Then, raw data were transferred from the mean Ct values of replicated samples to copy number of the established standard curve.

**Table 1:** Sequences of the primers used for qRT-PCR study.

**Gene symbol**	**Primer sequence (3′-5′)**	**Size (bp)**	**Accession no**
ACTB	F: CAT CAC CAT CGG CAA CGA	144	XM_003124280.5
	R: GCG TAG AGG TCC TTC CTG ATG T		
INSR	F: AGA GCG GAT CGA GTT TCT CA	245	XM_021083943.1
	R: CCA TCC CAT CAG CAA TCT CT		
PPARγ	F: ACA CCG AGA TGC CGT T	56	XM_005669788.3
	R: CGA CAG GTC CAC AGA G		
PPARA	F: CGA CCT GGA AAG CCC GTT AT R: GGA TCC ATC TGA TCC CGG AC	148	NM_001044526.1
ACVR1B	F: CAT CGA GGG GAT GAT CAA GT	211	NM_001195322.1
	R: GGC AAT GTC AAT GGT GTC AG		
CYP3A46	F: ACA GCA TTT GGA GTG AAC GTC	250	NM_001134824.1
	R: CCA CTC GGT GCT TTT GTG TAT		
GFPT1	F: ATG CTC TTC AGC AGG TGG TT	232	XM_005662490.3
	R: TCT ACG GTT ACC GAT TTG GC		
FFAR2	F: TCA TGG GTT TCG GCT TCT AC	191	XM_021093196.1
	R: AAC GAT GAA CAC GAC AGT GC		
TLR2	F: ACA TGA AGA TGA TGT GGG CC	109	XM_005653577.3
	R: TAG GAG TCC TGC TCA CTG TA		
TLR3	F: TAG AGA CAT GGA TTG CTC CC	435	NM_001097444.1
	R: AAC TTC TGG AAT GCA GGT CC		
TLR4	F: CTC TGC CTT CAC TAC AGA GA	322	NM_001293316.1
	R: CTG AGT CGT CTC CAG AAG AT		
EPCAM	F: GCG ATA GCG ATT GTT GCT GG	106	NM_214419.1
	R: CCC TAT GCA TCT CGC CCA TC		
SELL	F: GTG ATG CAG GGT ACT ACG GG	108	NM_001112678.1
	R: AGA ACT TGC CCA AAG GGT GA		
SAA2	F: AGA GCC TAC TCG GAC ATG AGA GA	65	NM_001044552.1
	R: CCC CGG GCA TGG AAG TAC		
CCL5	F: CCA GCA GCA AGT GCT CCA T	60	NM_001129946.1
	R: ACA CCT GGC GGT TCT TTC TG		
CXCL2	F: CCG GGA CCC CAC TGT GA	62	NM_001001861.2
	R: CAAACTTCCTGACCATTCTTGAGA		
CFB	F: CCT CGG GCT CCA TGA ATA TC	56	NM_001101824.1
	R: TGC CCC AAT GCT GTC TGA T		
C3	F: CCA ACA GGG AGT GCA ACG A	70	NM_214009.1
	R: TGA CTC CGT GTC TGG GAC TTG		
CSF1	F: CCA ACA GGG AGT GCA ACG A	147	NM_001244523.1
	R: TGA CTC CGT GTC TGG GAC TTG		
TGFB3	F: TTG CTA AAT GCT CCA GCC AG	90	NM_214198.1
	R: GCC TCC GCC TGT AGA ACA AG		
TNFAIP3	F: CCC TGG GGC ATT ATG GGT TT	60	NM_001267890.1
	R: CCT CAC ACG TTG TAG CAC CT		
CCL2	F: CCT CCC TGG AAA GCC AGA A	58	NM_214214.1
	R: GTG CCA CAA GCT TCC TCA CTT		
TNFα	F: CGA CTC AGT GCC GAG ATC AA	58	JF831365.1
	R: CCT GCC CAG ATT CAA AG		
IL8	F: GCT CTC TGT GAG GCT GCA GTT	62	NM_213867.1
	R: TTT ATG CAC TGG CAT CGA AGT T		
IL6	F: TGG ATA AGC TGC AGT CAC AG	109	NM_214399.1
	R: ATT ATC CGA ATG GCC CTC AG		
IL1α	F: AGA ATC TCA GAA ACC CGA CTG TTT	62	NM_214029.1
	R: TTC AGC AAC ACG GGT TCG T		
IL1β	F: GCC CTG TAC CCC AAC TGG TA	61	NM_001302388.2
	R: CCA GGA AGA CGG GCT TTT G		

*F, Forward; R, Reverse; bp, base pair*.

### Statistical Analysis of qRT-PCR Data

The raw data were log-transformed followed by checking the normality by Kolmogorov-Smirnov test. Comparisons between mean values were carried out using one-way ANOVA and Fisher's least significant difference test. For every cases, *p* < 0.05 was considered significant. The Pearson's correlation coefficient between the expression values obtained microarray and qRT-PCR were calculated to explore the linear relationship between the microarray and qRT-PCR results.

## Results

### Differentiation of Adipocytes From PIP

**Figure 1:**
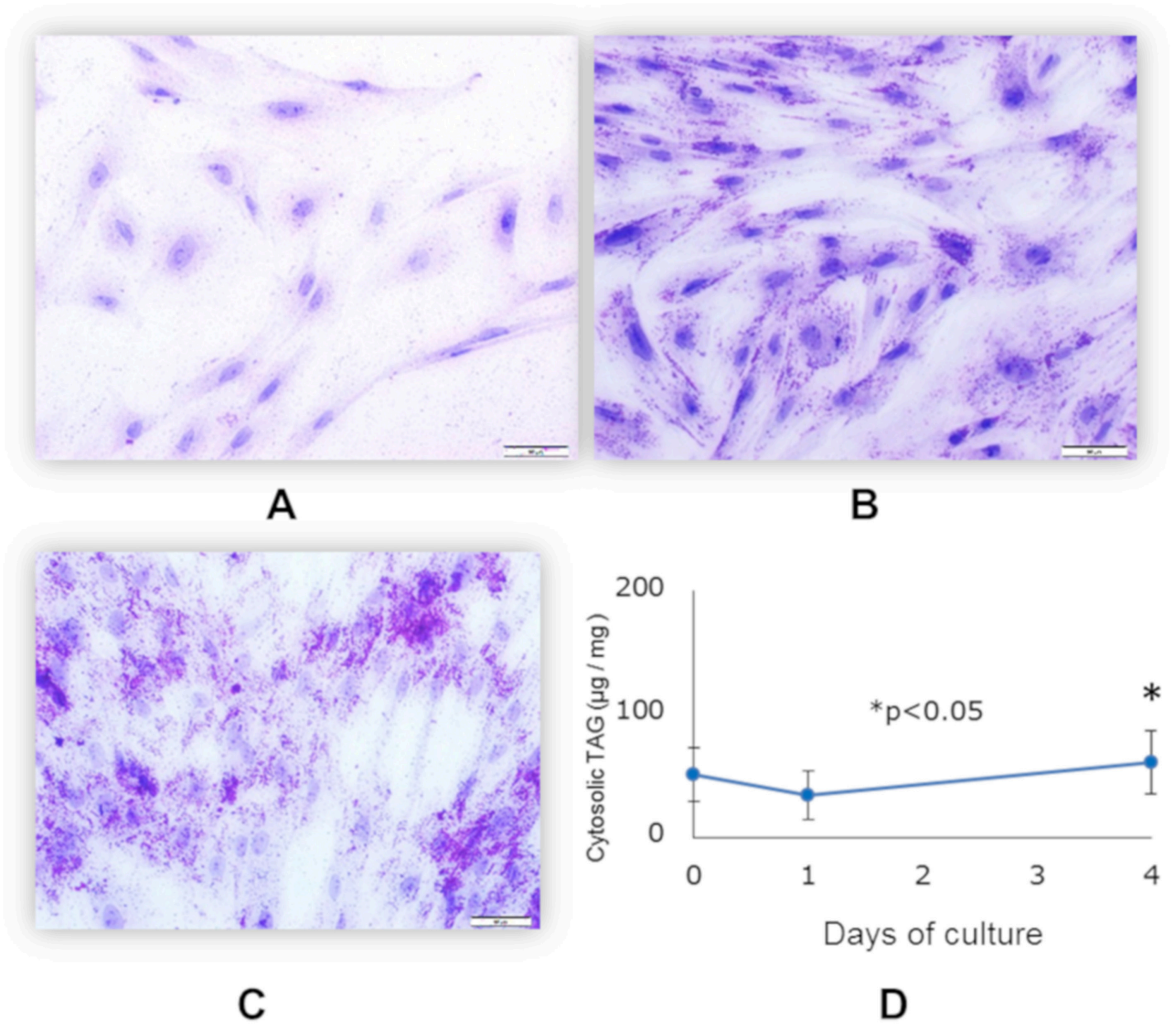
Differentiation of mature porcine adipocytes (pMA) from porcine intramuscular pre-adipocyte (PIP). Oil-red O stained images display the cellular morphology at day 0 **(A)**, day 1 **(B)**, and day 4 **(C)** of culture of PIP cells with differentiation media. Fat accumulation was determined by triglyceride assay **(D)**. Data (Mean ± SD) presented are the average of 3 independent experiments performed in triplicates.

Differentiation of functionally matured adipocytes from pre-adipocytes is an essential biological process. In order to obtain porcine mature adipocytes (pMA), we cultured the porcine intramuscular pre-adipocyte (PIP) cells (25) supplemented with differentiation medium for 4 days. The maturation of adipocytes was confirmed by the detection of lipid droplets in cell cytoplasm under microscopy using Oil red O staining as well as triglyceride assay. The PIP cells at the day of seedling showed no lipid droplets (Figure 1A). Following 1 day of culture, some cells started to exhibit flattened cellular morphology (Figure 1B). Finally, after 4 days of culture more than 95% of the PIP cells exhibited more lipid droplets indicated their differentiation into fully mature adipocytes (Figure 1C). A significant (*p* < 0.05) increased lipid accumulation within the cells indicated their maturation into adipocytes (Figure 1D).

**Figure 2:**
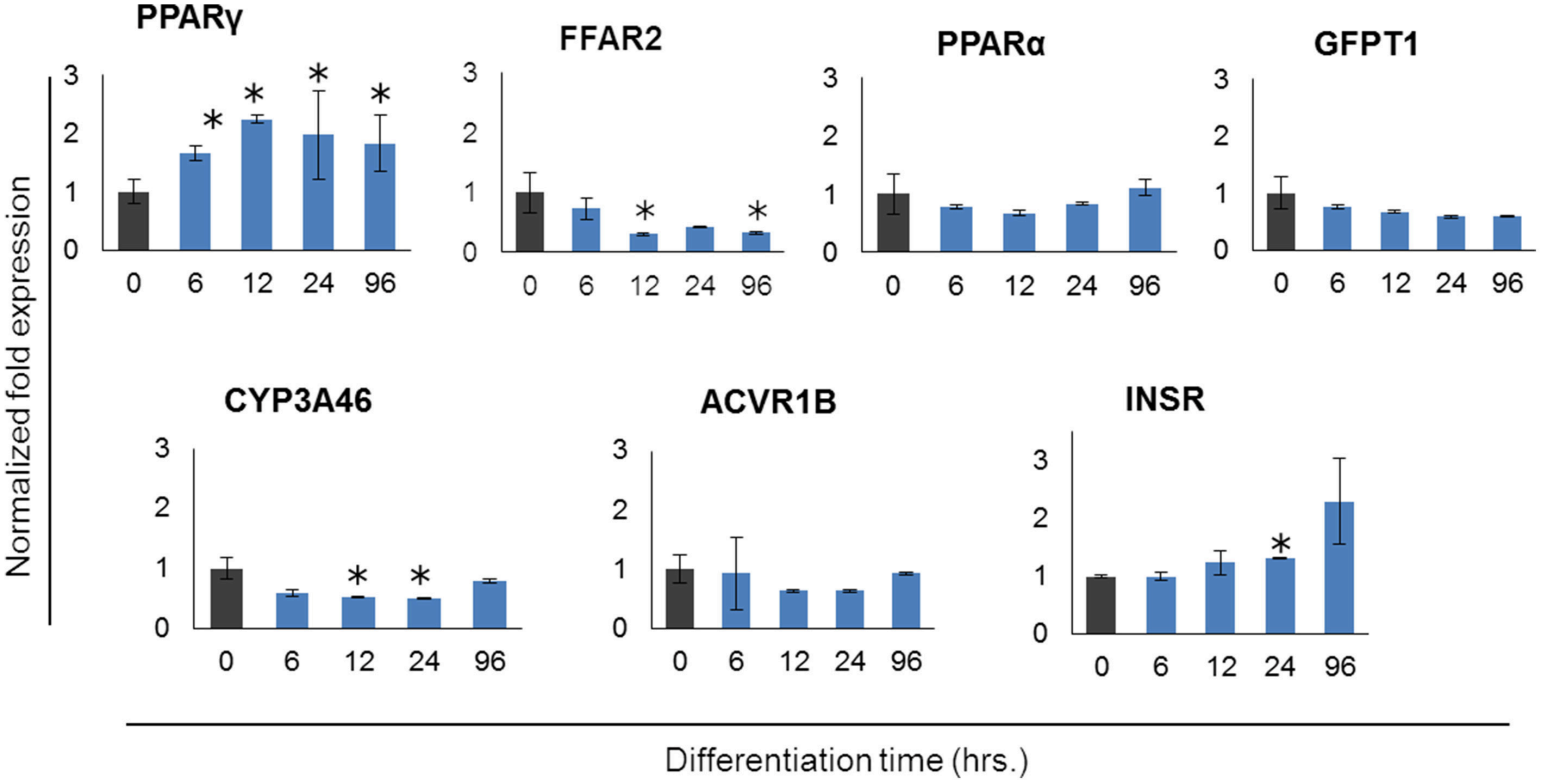
Expression profiles of marker genes for adipocyte differentiation in porcine intramuscular pre-adipocyte (PIP) and porcine mature adipocytes (pMA). The asterisk (^*^) indicates Statistical differences with significant levels of *P* < 0.05. Data (Mean ± SD) presented are the average of 3 independent experiments performed in triplicates.

In order to further confirm the adipocyte maturation, we quantified the expressions of some differentiation marker genes using qRT-PCR (Figure 2). The expression of peroxisome proliferator-activated receptor γ (PPARγ) and insulin receptor (INSR) were significantly increased in PIP after 4 days of culture with differentiation medium while cytochrome P540 receptor 3A (CYP3A46) and free fatty acid receptor 2 (FFAR2) were down regulated. The glutamine fructose-6-phosphate transaminase 1 (GFPT1) showed a decreasing trend but activin receptor type 1B (ACVR1B) and PPARα showed an increasing trend of expression (Figure 2).

### Differentially Expressed Transcripts in pMA After TLRs Activation

Stimulation of pMA with TLRs induced changes in a total of 1,575 genes. Among them, 702 (44.57%) and 873 (55.43%) genes were up- and down-regulated, respectively (Table 2). The TLR2 ligand Pam3csk4 differentially regulated 530 transcripts in pMA, being 218 and 312 transcripts up- and down-regulated, respectively. After TLR3 stimulation 245 transcripts were up-regulated while 275 transcripts were down-regulated in pMA. In addition, stimulation of pMA with LPS up-regulated 239 transcripts and down-regulated 286 transcripts (Table 2). The list of significantly (adjusted p<0.05) up-regulated and down-regulated genes are presented in Supplementary Tables S1–S3.

**Table 2:** Number of differentially regulated genes in the porcine intramuscular adipocytes after Pam3csk4, Poly(I:C) and LPS stimulation.

	**Pam3csk4 vs. control**	**Poly(I:C) vs. control**	**LPS vs. control**
Up	218	245	239
Down	312	275	286
Total	530	520	525

### Intersections of TLR Ligand-Induced Differentially Regulated Transcripts

**Figure 3:**
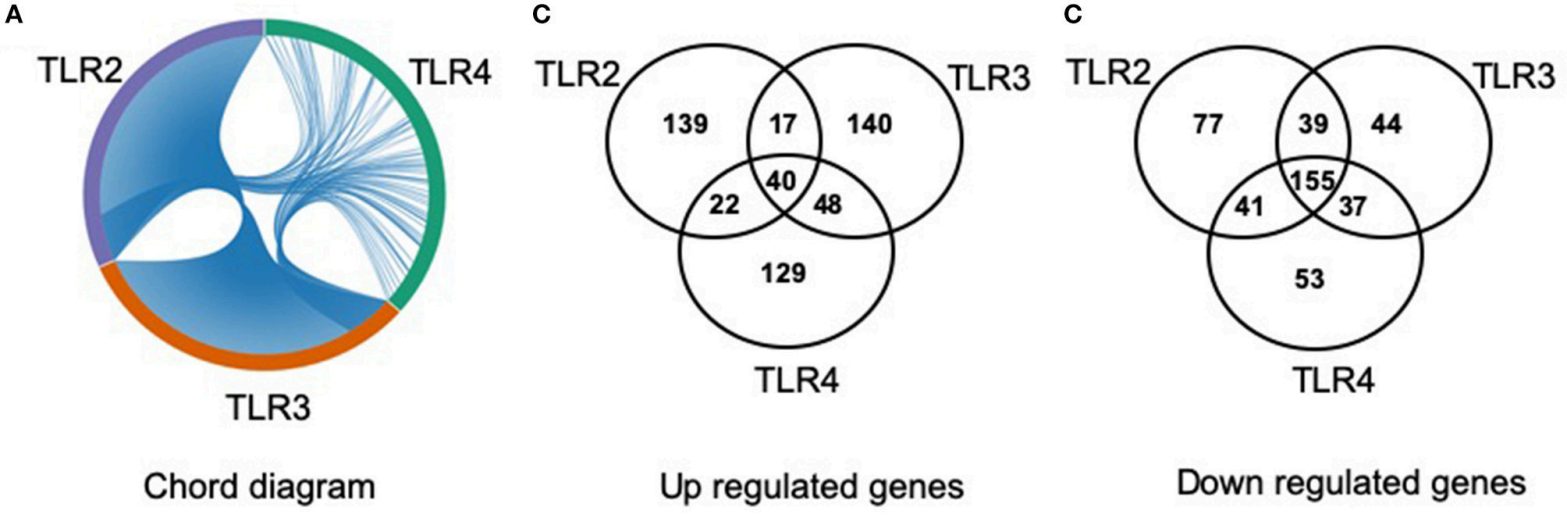
Distribution of number of differentially regulated genes in mature porcine adipocytes (pMA) among intersections of three contrast pairs of ligand stimulation. **(A)** Chord diagram showing the proportion of transcripts shared among stimulants. **(B)** The number of up regulated transcripts after each stimulant and their intersections. **(C)** The number of down regulated transcripts after each stimulant and their intersections.

The lists of differentially regulated transcripts obtained from three pairwise contrasts were overlapped to assess the cross-talk among the three TLR ligands. We observed that there were 195 differentially expressed transcripts shared by the three stimulations. Among them, 40 were up- and 155 were down-regulated transcripts (Figure 3). A total of 251 differentially expressed transcripts were common between TLR2 and TLR3 stimulation, 57 and 194 were up- and down-regulated, respectively. In addition, 280 differentially expressed transcripts were common between TLR3 and TLR4 stimulation. A total of 258 transcripts were shared by pMA stimulated with TLR2 or TLR4. Among them, 62 were up- and 196 were down-regulated transcripts (Figure 3).

### Gene Ontology Classification

**Figure 4:**
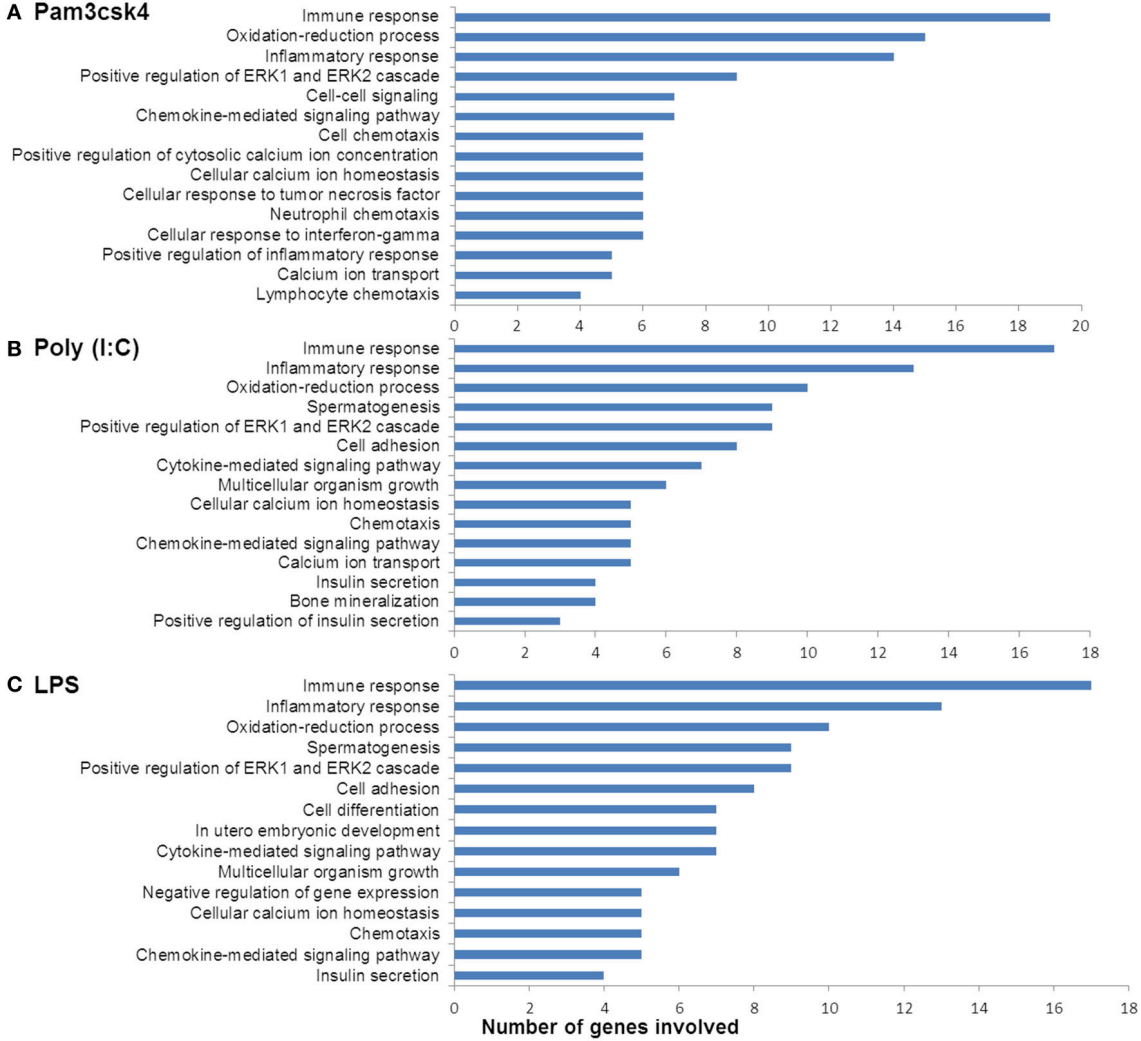
Enrichment of GO terms by differentially expressed genes in porcine mature adipocyes (pMA) after stimulation with **(A)** Pam3csk4, **(B)** Poly(I:C), and **(C)** LPS. GO terms shown were passed the statistical significance threshold (*p* < 0.05).

In order to characterize the biological implications of the differentially expressed genes in pMA after TLRs stimulations, we performed the GO and pathway enrichment analysis using DAVID online tool. Based on the ascending order of adjusted *p*-value, the top 15 GO biological process significantly enriched with the differentially expressed genes following Pam3csk4, Poly(I:C), and LPS stimulation are presented in Figure 4. There were some common GO terms enriched following each of the three TLR treatments including immune response (GO: 0006955), inflammatory responses (GO: 0006954), oxidation-reduction process (GO: 0055114), chemokine-mediated signaling (GO: 0070098), and positive regulation of ERK1 and ERK2 cascade (GO: 0070374). Cell adhesion (GO: 0007155), chemotaxis (GO: 0006935), and insulin secretion (GO: 0030073) were commonly enriched GO biological process between Poly(I:C) and LPS stimulation, while enrichment of calcium ion transport (GO: 0006816) was shared between Pam3csk4 and Poly(I:C) stimulation. The full list of enriched GO and pathways are presented in Supplementary Table S4.

### Transcript Abundances Associated With Immune Response Function

**Figure 5:**
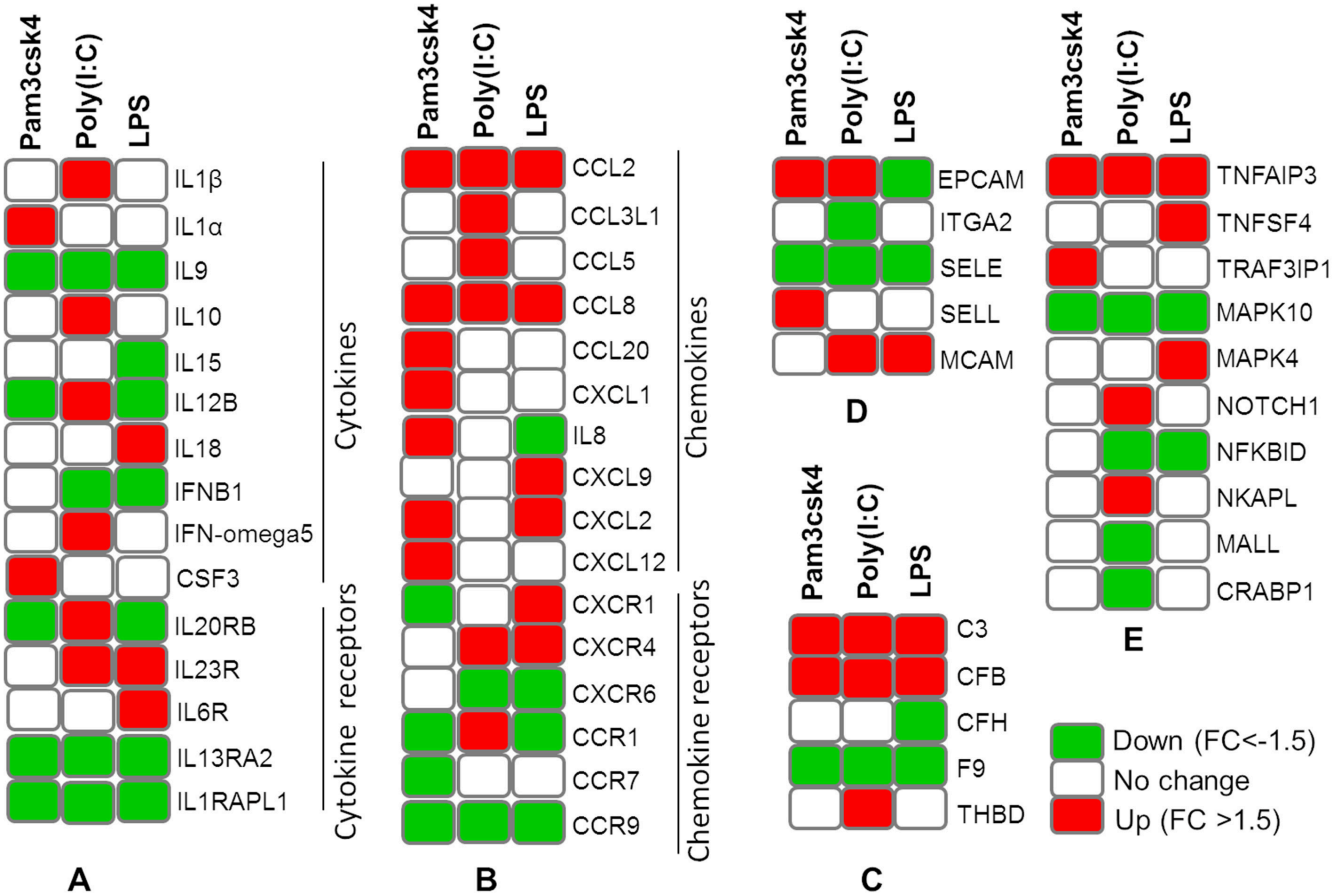
Heatmap showing the expression patterns of different groups of immune related genes measured by microarray in mature porcine adipocytes (pMA) stimulated with different TLRs ligands. Groups of genes include cytokines and cytokine receptors **(A)**, chemokines and chemokine receptors **(B)**, complement factors **(C)**, adhesion molecules **(D)**, and signal transduction molecules **(E)**.

The expression patterns of differentially modulated immune related genes in pMA following TLR activation are presented in Figure 5. The mRNAs of cytokine and cytokine receptors including *IL1*β*, IL1*α*, IL13RA2, IL1RAPL1, IL9, IL10, IL15, IL6R, IL12B, IL18A, IL20RB, IL23R, IFNB1*, and *IFN-omega5* showed differential expression. The expression of chemokine and chemokine receptors including *CCL2, CCL20, CCL8, IL8, CX3CR1, CXCL9, CSF3, CXCL12, CXCR4, CCL5, CCL3L1, CCR1, CCR7, CXCR6, CCR9*, and *CXCL1* were differentially regulated. The expression of complement factors including *C3, CFB, CFH, F9*, and *THBD*; and adhesion molecules including *EPCAM, ITGA2, SELE, SELL*, and *MCAM* were differentially modulated following TLR activation in pMA. The signal transduction molecules including *TNFAIP3, TNFSF4, TRAF3IP1, MAPK, NOTCH1, NF-kB, NKAPL, MALL*, and *CRABP1* showed differential expression in pMA after 12 h of TLR activation (Figure 5).

### Transcripts Abundance Associated With Metabolic and Endocrine Functions

**Figure 6:**
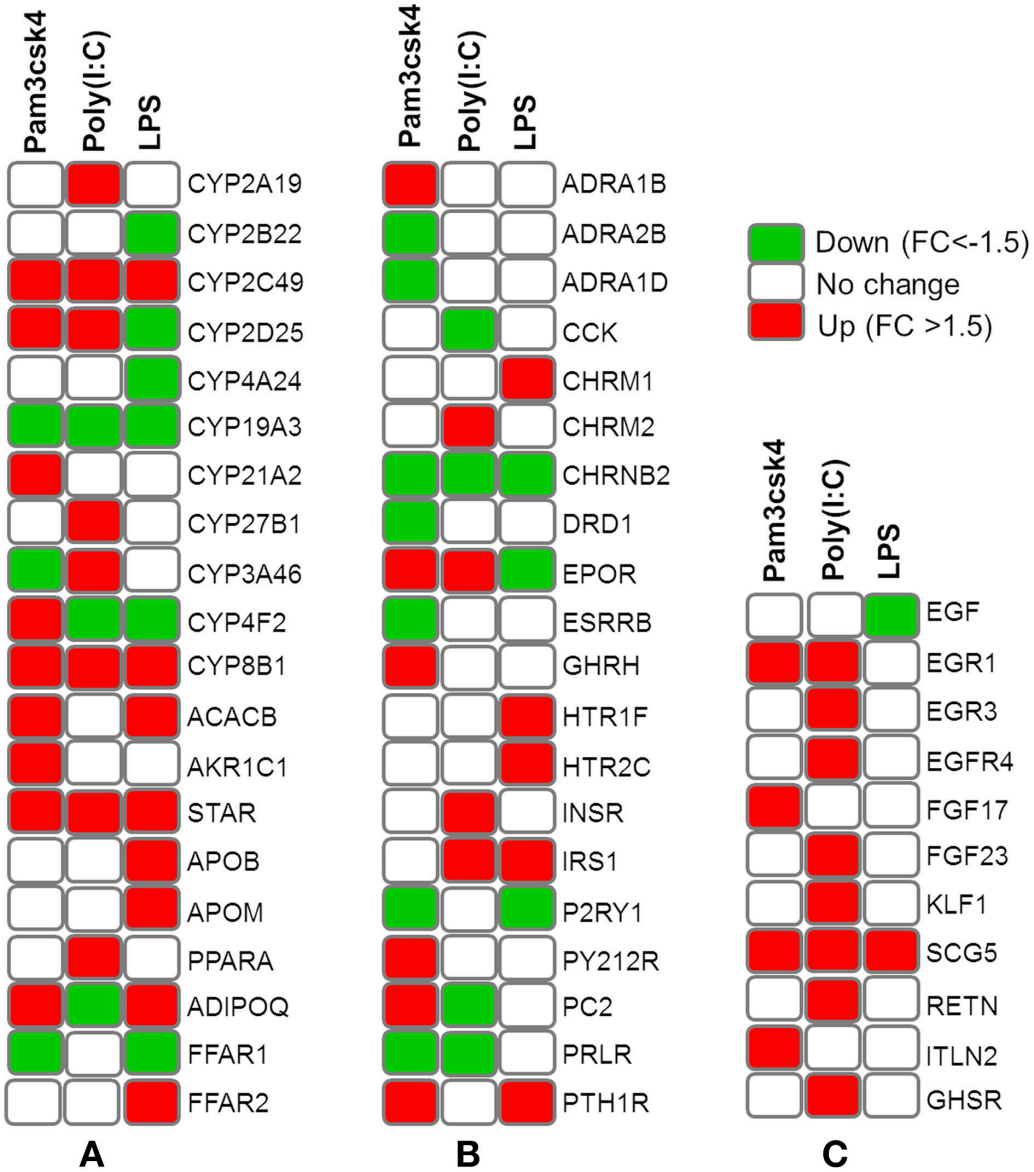
Heatmap showing the expressions of genes associated with metabolism and endocrine functions measured by microarray in mature porcine adipocytes (pMA) stimulated with different TLRs ligands. Groups of genes include those related to lipid metabolism **(A)**, hormones and receptors **(B)**, and growth factors **(C)**.

Differentially regulated transcripts encoded for proteins involved in metabolism and endocrine functions are summarized in Figure 6. Among them, genes involved in lipid metabolism included *FFAR2, APOB, APOM, ADIPOQ, CYP8B1, CYP2C49, CYP2025, STAR, CYP21A2, ACACB, AKR1C1, CYP4A24, CYP2B22, FFAR1, PPARA, CYP27B1, CYP3A46, CYP2A19, CYP4F2*, and *CYP19A3*. The mRNAs for hormones and receptor including *ADRA1B, ADRA2B, ADRA1D, CCK, CHRM2, CHRNB2, DRD1, EPOR, ESRRB, GHRH, HTR1F, HTR2C, INSR, IRS1, P2RY1, P2Y12R, PC2, PRLR*, and *PTH1R* showed differential expressions in pMA after TLR activation. In addition, TLR activation resulted in a differential expression of some growth factors including *EGF, EGR1, EGR3, EGFR4, EGF17, EGF23, KLF1, SCG5, RETN, ITLN2*, and *GHSR* in the porcine adipocytes (Figure 6).

### Gene Expression Measured by qRT-PCR

**Figure 7:**
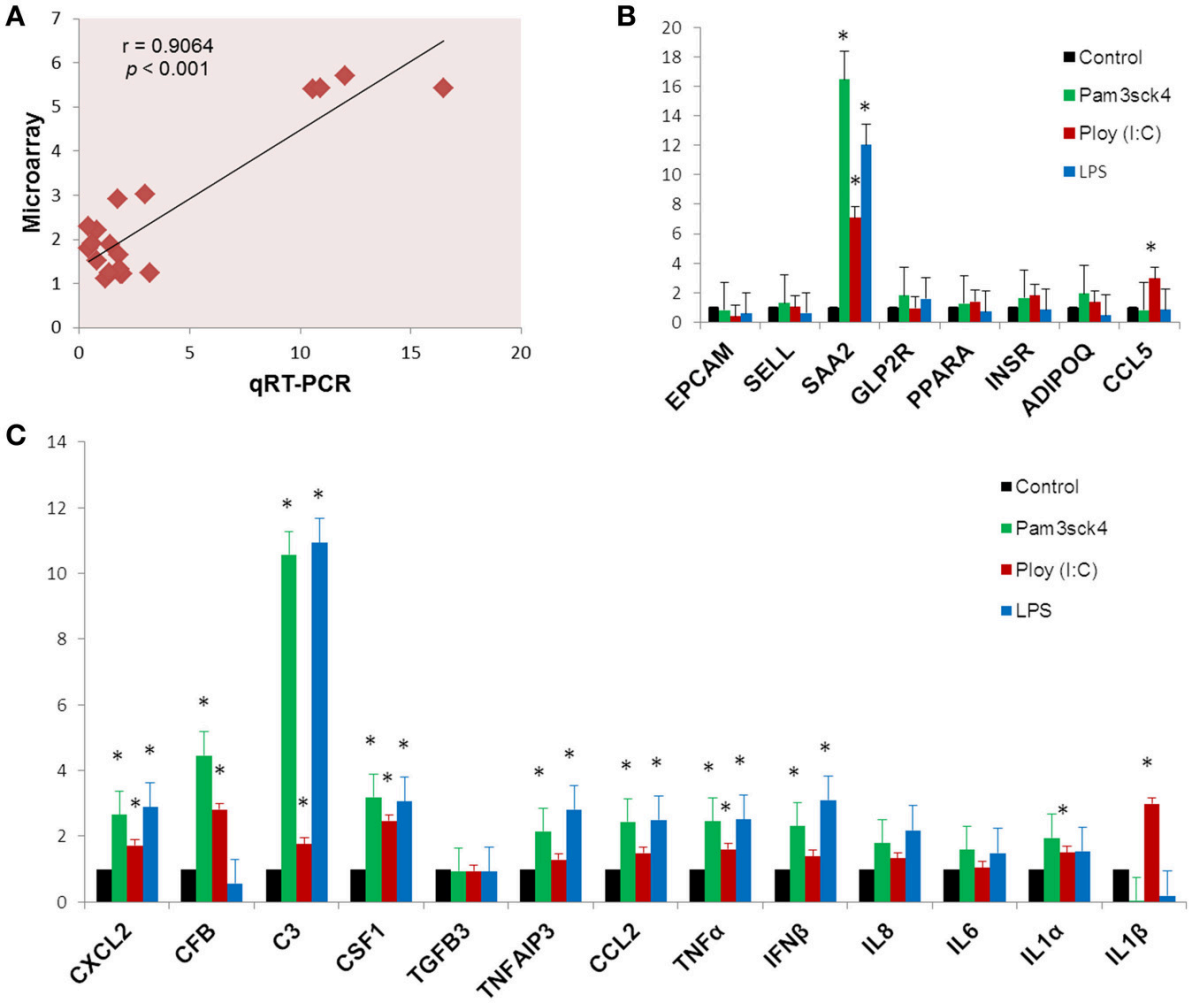
Gene expression results obtained from qRT-PCR. The Pearson's correlation coefficient between expression results obtained from microarray and qRT-PCR **(A)**. The expression profiles of metabolism **(B)** and immune response **(C)** related genes measured by qRT-PCR. Y-axis represents the fold expressions. The asterisk (^*^) indicates Statistical differences with significant levels of *p* < 0.05. Data (Mean ± SE) presented are the average of 3 independent experiments performed in triplicates.

In order to confirm the microarray expression results, we quantified mRNAs of several differentially expressed genes as well as some other genes which are known to be involved in immune and metabolic functions by using qRT-PCR. The Pearson's correlation coefficient (*r* = 0.9064, *p* < 0.001) indicated that microarray expression results were strongly correlated with that obtained from qRT-PCR (Figure 7A). Among the genes quantified by qRT-PCR, *SAA2* was significantly up regulated and *EPCAM* was down regulated after all the ligand stimulation (Figure 7B). The expression of *SELL, PPARA, INSR*, and *ADIPOQ* were up regulated after both Pam3csk4 and Poly(I:C) stimulation but down regulated after LPS stimulation. *GLP2R* expression was increased after Pam3csk4 and LPS stimulation but *CCL5* was increased only after Poly(I:C) stimulation (Figure 7B). Among the immune response related genes, up regulation of *CXCL2, C3, CSF1, TNFAIP3, CCL2, TNF*α*, IFN*β*, IL8, IL6*, and *IL1*α were noticed relatively more in case of Pam3csk4 and LPS stimulation than Poly(I:C) stimulation (Figure 7C). The expression of CFB was increased after Pam3csk and Poly(I:C) stimulation but decreased after LPS stimulation. *IL1*β was increased after Poly(I:C) stimulation, but decreased after Pam3csk4 and LPS stimulation. Expression of *TGFB3* was remained stable following all three stimulation (Figure 7C).

### Protein-Protein Interaction (PPI) Network

**Figure 8:**
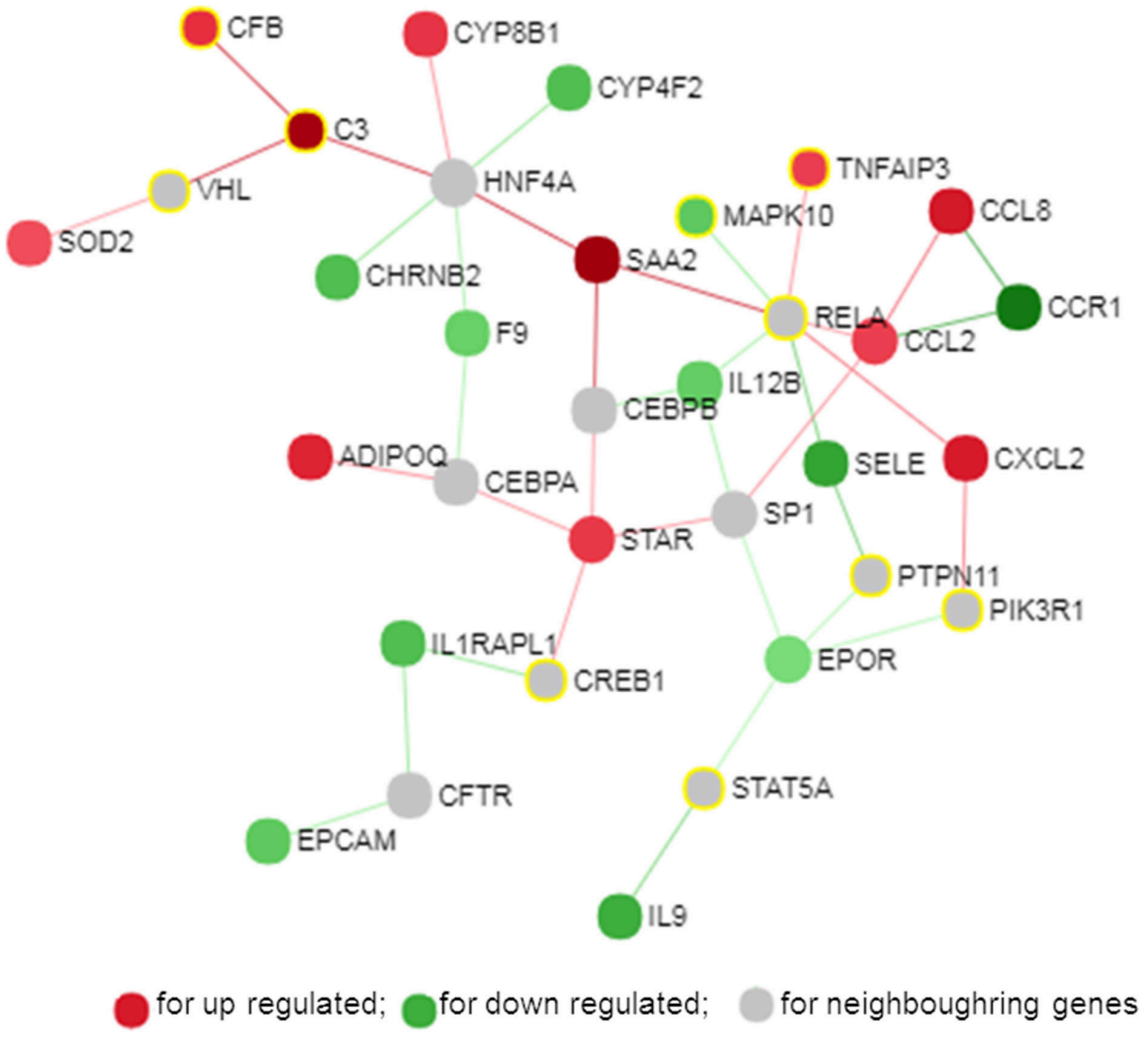
Protein-protein interaction network of the common genes in mature porcine adipocytes (pMA) stimulated with different TLRs ligands obtained from NetworkAnalyst.

The PPI network is a hierarchical structure, where the hubs play a central role in directing cellular response to a given stimulus. To identify the hub genes involved in the regulation of transcriptome modification of pMA following TLRs ligation, we have constructed and visualized the PPI network of differentially expressed transcripts (Figure 8). The hub nodes of the network were selected based on the values of two centrality measures interpreted serially, first “degree” then “betweenness.” Accordingly, *RELA, HNF4A, SP1, EPOR, C3, STAR, CCL2*, and *SAA2* were identified as major hub genes of the TLRs induced transcriptional network in the pMA (Figure 8). Though *RELA, HNF4A*, and *SP1* were not differentially expressed in the TLR ligand-treated pMA but predicted to be involved in the regulation of PPI-network. The centrality measures of the network nodes/interactome are presented in Supplementary Table S5.

### Gene-Centered Transcription Factor Network

**Figure 9:**
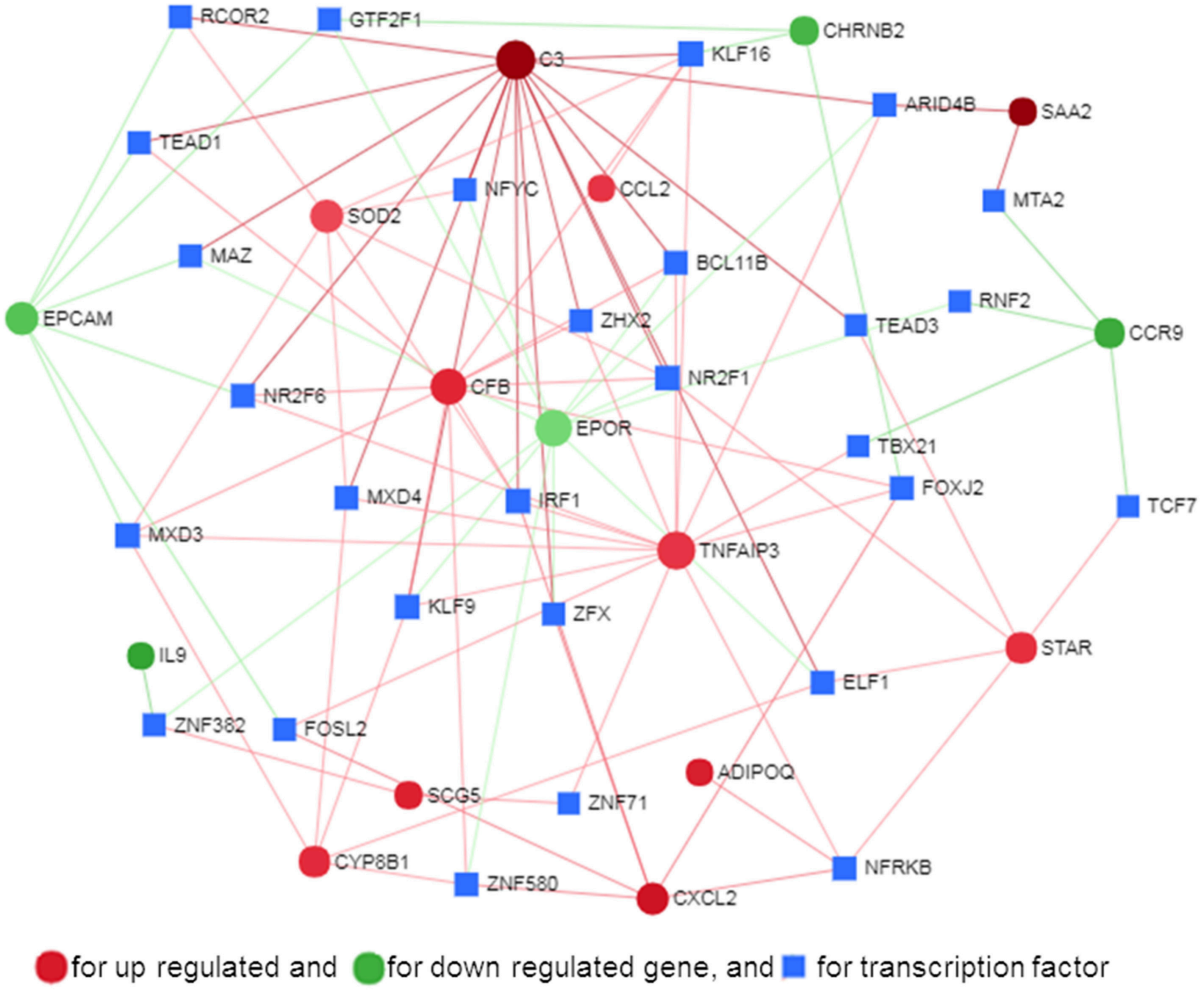
Gene-transcription factor regulatory network for the common genes in mature porcine adipocytes (pMA) stimulated with different TLRs ligands obtained from NetworkAnalyst.

Transcription factors (TF) are potential regulators of differential gene expression. We constructed a gene-TF interaction network in order to explore the involvement of transcription factors in TLR ligand-induced differential gene expression in porcine adipocytes using NetworkAnalyst. Gene-TF network illustrated that potential hub genes of PPI network also exhibited higher centrality estimates in the Gene-TF interaction network indicating their possible regulation by transcription factors. The transcriptional factor binding site (TFBS) analysis revealed that promoter regions of C*3, TNFAIP3, CFB, CXCL2, STAR, ADIPOQ, CYP8B1, SOD2, EPCAM, EPOR, SAA2*, and *CCR9* genes have DNA-binding sites of transcription factors, which are likely contributing pMA transcriptional modification (Figure 9). In particular, *TNFAIP3, CXCL2, STAR*, and *ADIPOQ* are regulated by *NF-kB* transcription factor. The network centrality measures of potential genes are presented in Supplementary Table S6.

## Discussion

In recent years, it became clearer that along with the metabolic and endocrine functions, the adipose tissue exerts multiple roles in the generation and regulation of immune responses (4, 13). In this work, we performed a microarray-based global transcriptome profiling of pMA after *in vitro* stimulation with TLR ligands in order to evaluate the immune and metabolic responses of porcine adipocytes triggered by the activation of these PRRs.

The differentiation and maturation of adipocytes is crucial for their proper physiological functions and for the prevention of metabolic disorders (36, 37). Apart from those in the main subcutaneous and visceral fat depots, adipogenic differentiation has also been described in other locations, including skin, bone marrow, and skeletal muscle (36). A recent microarray study based on primary cells of intramuscular preadipocytes obtained from Landrace pigs has reported gene expression changes associated with adipocyte differentiation (38). Among other genes, pattern recognition receptors were differentially expressed when preadipocytes were compared with pMA (38), which was in line with our previous findings (28). Then, we focused our attention in pMA. Here, we confirmed the differentiation of intramuscular preadipocytes cell line obtained from Duroc pigs by the evaluation of fat accumulation, and expression of several marker genes. Thus, adipocytes used for this transcriptome analysis were functionally mature. In addition, in our previous study we demonstrated that members of the TLR family including TLR2, TLR3, and TLR4 focused in the present study are expressed in pMA (28).

**Figure 10:**
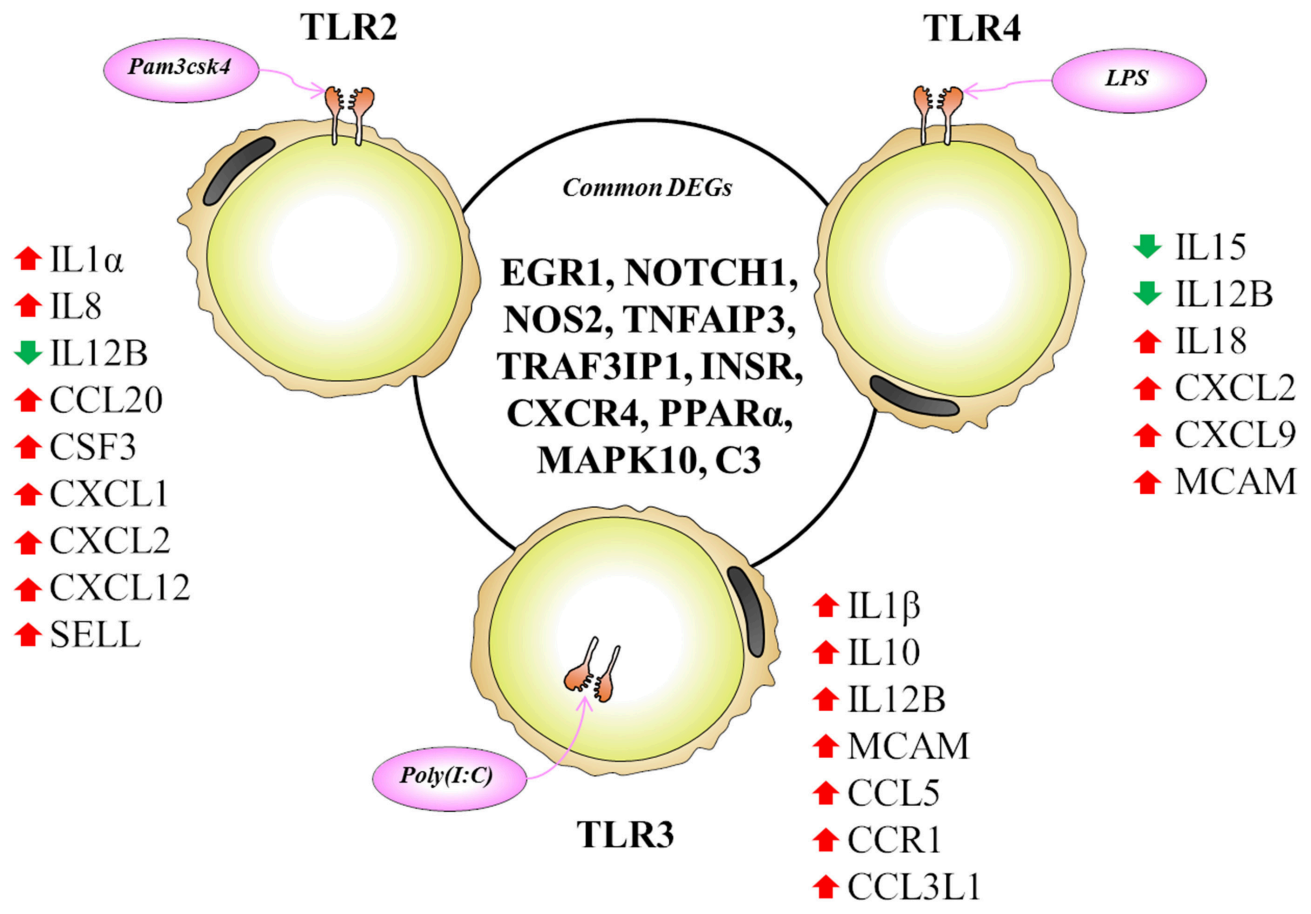
Most significantly altered genes after TLR2, 3 and 4 activation in porcine adipocytes. Genes presented in central circle are the common DEGs for all three TLR activation while individual list close to each TLR are the unique DEGs for them. Red arrow indicates upregulation while green arrow indicates the downregulation of corresponding genes.

The TLR2 is activated by the lipopeptides/peptidoglycan present in the cell wall of bacteria. It has been reported that human and murine adipocytes express TLR2 (15, 25, 39). Stimulation of adipocytes from humans and mice with the TLR1/2 ligand Pam3Csy or the TLR2/6 ligand MALP-2 differentially modulated the release of the proinflammatory factors including *IL6, IL8*, and *CCL2* in those cells (15, 39), whereas resistin was either not affected or even down regulated by both ligands (15). The role of the adipose tissue in the innate immune response induced by local or systemic infection with *Staphylococcus aureus* has been studied *in vivo* in a rodent model (40). The work reported that systemic bacterial infection resulted in a shift from anti- to pro-inflammatory cytokines in the adipose tissues transcriptome profile clearly demonstrating the role of adipocytes in the development of immune response (40). To the best of our knowledge, no transcriptional analysis has been performed in porcine adipocytes in response to TLR2 activation. Similar to human and murine adipocytes, we observed that pMA stimulated with Pam3csk showed a major transcriptome alteration, and that the differentially regulated transcripts were known to be involved in the positive regulation of inflammatory responses, including cytokines (*IL1*α*, IL12B*), chemokines (*CXCL1, CXCL2, CCL20, CSF3*), and adhesion molecules (*SELL*) (Figure 10). This result indicates the ability of porcine adipocytes in participating in immune responses triggered by TLR2 activation.

On the other hand, the LPS from gram-negative bacteria is a well-known innate immune stimulant of TLR4 activation (6). The responsiveness of human adipocytes to LPS stimulation has been widely documented (15, 25); therefore, we speculated that porcine adipocytes would also have the ability to response to LPS in a similar way. LPS/TLR4 interaction induces signaling pathways through the adaptor molecule MyD88, which results in the activation of transcription factor such as NF-kB and AP-1 (6, 9, 41). MyD88 recruits different interleukin-1 receptor associated kinase (IRAK) family proteins and TNF receptor-associated factors 6 (TRAF6) (42). This complex activates TGF-activated kinase 1 (TAK1), leading to activation of NF-kB and mitogen activated protein kinases (MAPKs) (43), which in turn induce the expression of factors participating in inflammatory responses. In agreement, we observed that several signal transduction molecules including members of TNF, MAPK, and NF-kB pathways were found to be differentially expressed in pMA following the activation of TLR4. Of note, pMA stimulated with LPS showed an up-regulation of *CXCL2* and *CXCL9* that are strongly chemotactic for lymphocytes. Similar to our results, LPS up-regulated the expression of *CCL2* and *CCL8* in human adipocytes (44), and *CCL2* in mouse adipocytes (15). Of note, although microarray results showed down-regulation of *IL8* and no effect on *IL6* expression after LPS stimulation (Figure 5), we checked the expression of both cytokines by qRT-PCR because of their up-regulation is well-reported in LPS responses of human (44), mouse (15), and pig (45) adipocytes. Although it was statistically insignificant, but both *IL6* and *IL8* expression showed an upward trend after LPS stimulation (Figure 7C). In addition, an increase in the expression of *IL18*, which is a cytokine belongs to the *IL1* superfamily and has the ability to stimulate the cellular immune response through the activation of natural killer cells (NK) cells and T cells, was observed after TLR4 activation in pMA (Figure 10). These results are in line with study of Vielma et al. (46) who used fibroblast-derived adipocytes and spleen lymphocytes in order to evaluate whether adipose cells were able to modulate the function of immune cells. The work demonstrated that adipocyte-conditioned medium was able to activate spleen lymphocytes and stimulate their production of the inflammatory cytokines IL6, IL9, IFNγ, and TNFα.

Interestingly, TLR3 activation in pMA also led to the development of a complex transcriptomic response. TLR3 ligation lead to the activation of NF-kB and MAPKs to induce pro-inflammatory cytokines (43), and the phosphorylation and activation of IRF3, leading to IFNγ (42) and type I interferons (47) production. It was described that TLR3 is highly expressed and functionally active in human (48) and mouse (49) adipocytes. Stimulation of human and mouse adipocytes with Poly(I:C) is able to induce the expression of pro-inflammatory factors such as *TNF*α, *IL6, IL8*, and *CCL2* as well as IFN-α/β and multiple anti-viral proteins including 2′5′-oligoadenylate synthetase and Mx GTPase 1 (48, 49), indicating that adipose cells are able to trigger innate antiviral responses. Here, we observed that TLR3 activation in pMA increased the expression of several inflammatory genes (*CCL2, CCL8, CCL5, CCL3L1, IL1*β*, IL12B*, and *MCAM*) that participate in the antiviral inflammatory responses resembling human and mice adipocytes (Figure 10). However, our transcriptomic analysis was no able to detect increases in the expression of IFN-α/β or IFN-induced antiviral genes. Deeper kinetic and dose-response studies are necessary to establish whether this difference between porcine and human/mice adipocytes are due to differences related to the species or are an effect of the experimental conditions.

Activation of human adipocytes by multiple TLRs ligands was reported to induce pro-inflammatory and pro-diabetic responses through the phosphorylation of extracellular signal-induced kinase and c-Jun N-terminal kinase pathways (25). Human adipocytes have shown the ability to synthesize and secrete complement factors, C1q/TNF-related proteins (CTRPs) (15, 16), cytokines, chemokines, and pro- and anti-inflammatory adipokines including resistin, visfatin, leptin, and adiponectin; as well as antibacterial peptides such as lipocalin-2 and cathelicidin in response to multiple TLRs ligands (25, 50). In addition, taking into consideration that in most cases microorganisms carry more than one TLR-ligand and that infections with multiple pathogens, such as virus-bacteria superinfections, have become a common disease scenario; we focused in the transcripts showing differential expression after the activation with the three TLRs in pMA. These commonly differentially regulated genes (Figure 10) are involved in two major biological functions: inflammatory response and insulin mediated metabolism, in agreement with previous reports that evaluated TLRs activation in human adipocytes (15, 16, 25, 48). In fact, immune response, cytokine-mediated signaling, inflammatory responses, oxidation-reduction process, positive regulation of ERK1/ERK2 and lipid metabolisms are commonly enriched GO terms in pMA following TLR2, TLR3, or TLR4 activation.

Here we report that *EGR1, NOTCH1, NOS2, TNFAIP3, TRAF3IP1, INSR, CXCR4, PPARA, MAPK10*, and *C3* are the top 10 (based on fold expression) commonly altered genes of TLRs induced transcriptional modification in pMA (Figure 10). It is well-known that *TNFAIP3, CXCR4, MAPK4, MAPK10*, and *IL8* among others are involved in the generation of innate immune responses (2, 6, 8, 9). Other common DEGs like *EGR1* and *INSR* are known to be involved in insulin and glucose metabolism (35). *PPARA* and *NOS2* participate in adipocyte maturation (35, 37). However, the immune response and the lipid metabolism are complex traits, not regulated only by a particular gene but rather by networks of complex molecular interactions (51). Therefore, network analysis based on larger immune-specific and metabolic-specific gene databases is considered to be more effective strategy for identification of regulatory genes (31). The PPI network predicted *RELA, HNF4A, SP1, EPOR, C3, STAR, CCL2*, and *SAA2* as the major regulatory hub genes of TLRs-induced transcriptional network in porcine adipocytes. Interestingly, *C3* is the hub gene that overlapped with top 10 common DEGs. The *C3* is the central component of complement system and plays a vital role in the innate immune response, which was up-regulated in pMA stimulated with TLRs ligands. It was reported that the agonists for TLR2, TLR4, and TLR9 are able to activate the complement-TLR crosstalk, which signals through MyD88 pathway and mediated inflammatory responses (52). We also observed that the hub genes of PPI network also exhibited higher centrality estimates in the gene-centered transcription factor interaction network indicating their potential to regulate the TLR-induced transcriptional modifications in the porcine adipocytes. A long-term goal of our laboratory is to establish an adipocyte-based *in vitro* evaluation system for the selection of beneficial microbes able to differentially modulate immune responses in the adipose tissue for their application in the prevention of immunometabolic diseases in human and animals. We have been successful in a similar approach before by establishing a porcine intestinal epithelial (PIE) cells model for the efficient selection of anti-diarrheal immunomodulatory probiotic bacteria (53).

The physiological roles including metabolic and secretory function of adipocytes are distinguished by their tissue origin, appearance and location of the body (54, 55). The intramuscular adipocytes play pivotal role in the control of systemic energy balance (56) and immuno-metabolic homeostasis (57). However, due to their particular location in close vicinity with muscle fibers, the biology of intramuscular adipocytes may differ from those originated from other location. Among the hub genes identified in this study, *EPOR, STAR*, and *SAA2* are known to be involved in lipid metabolism. Serum amyloid A (SAA) including *SAA2*, have been reported to function in metabolic homeostasis and healthy adipose development through accompanying with retinoic acid, a potential regulator of lipid metabolism (58). In addition, though the lipolysis pathway was not induced after TLRs ligation in intramuscular derived pMA, the intracellular calcium homeostasis and positive regulation of insulin secretion pathways were significantly enriched, which indicates that the TLR-induced transcriptional modification is linked to metabolic changes. These are in line with finding of Gardan et al. (55) who reported that porcine intramuscular adipocytes display a relatively lower lipogenic activity compared with adipocytes isolated from subcutaneous or visceral adipose tissue. Besides the subcutaneous and visceral fat depots, adipogenic differentiation appears to be beneficial in the skin; however, adipogenesis in the skeletal muscle is associated with pathology (36). Therefore, the results obtained in this work indicate that pMA are a useful tool for the *in vitro* study of the transcriptomic changes associated not only with the innate immune response, but also with inflammatory disorders linked to lipid metabolism and hormones resulting from TLRs ligation in porcine adipocytes.

## Conclusion

This study reported for the first time the global transcriptome modifications in porcine adipocytes following activation of TLR2, TLR3, or TLR4. We demonstrated that the activation of TLRs in porcine mature adipocytes induced modifications of transcripts involved not only in the immune response, but also in cellular lipid metabolism. Sub-network enrichment analysis suggested that *EPOR, C3, STAR, CCL2*, and *SAA2* are the major hub genes of TLRs-mediated transcriptional network responses of porcine adipocytes. In addition, gene-transcription factor interaction network analysis revealed the potentials of hub genes to regulate the TLRs-induced transcriptional modification. Therefore, we identified key regulatory genes that could be used as candidates for the evaluation of immune-modulators (e.g., immunomodulatory probiotic bacteria) having functional beneficial effects (e.g., anti-inflammatory capacity) in porcine adipocytes.

## Data Availability

The MIMAE (minimum information about a microarray experiment) standard raw microarray dataset have been submitted to the NCBI-GEO database under the access number GSE124171.

## Author Contributions

MI, MAI, JV, and HK designed the study. MI, AT, and MT did the cell culture and qRT-PCR experiments, and prepared the microarray samples. KM, KY, and FH arranged the kit, chemicals, and reagents. MAI analyzed the expression data and wrote the manuscript. LA contributed to data analysis and results interpretation. AK, WI-O, HA, and HT reviewed the manuscript. HK and JV approved the final version of the manuscript.

### Conflict of Interest Statement

KM, KY, and FH are employed by Takanashi Milk Products Co., Ltd. The remaining authors declare that the research was conducted in the absence of any commercial or financial relationships that could be construed as a potential conflict of interest.

## Supplementary Material

Supplementary Table S1 Top 40 significantly (adjusted *p* < 0.05) differentially expressed genes in pMA after Pam3csk stimulation (.xlxs).

Supplementary Table S2 Top 40 significantly (adjusted *p* < 0.05) differentially expressed genes in pMA after Poly(I:C) stimulation (.xlxs).

Supplementary Table S3 Top 40 significantly (adjusted *p* < 0.05) differentially expressed genes in pMA after LPS stimulation (.xlxs).

Supplementary Table S4 GO terms and pathways enriched by TLR ligand-induced DEGs in adipocytes obtained from DAVID analysis (.xlxs).

Supplementary Table S5 Centrality estimates of protein interaction network of TLR ligand-induced DEGs in adipocytes obtained from NetworkAnalyst (.xlxs).

Supplementary Table S6 Centrality estimates of Gene-transcription factor interaction network of TLR ligand-induced DEGs in adipocytes obtained from NetworkAnalyst (.xlxs).

## References

[1] Akira S Uematsu S Takeuchi O. Pathogen recognition and innate immunity. Cell. (2006) 124:783–801. 10.1016/j.cell.2006.02.015 16497588

[2] Kawai T Akira S. Toll-like receptors and their crosstalk with other innate receptors in infection and immunity. Immunity. (2011) 34:637–50. 10.1016/j.immuni.2011.05.006 21616434

[3] Chen GY Nunez G. Sterile inflammation: sensing and reacting to damage. Nat Rev Immunol. (2010) 10:826–37. 10.1038/nri2873 21088683 PMC3114424

[4] Grant RW Dixit VD. Adipose tissue as an immunological organ. Obesity. (2015) 23:512–8. 10.1002/oby.21003 25612251 PMC4340740

[5] Piccinini AM Midwood KS. DAMPening inflammation by modulating TLR signalling. Mediat Inflamm. (2010) 2010:672395. 10.1155/2010/672395 20706656 PMC2913853

[6] Kawai T Akira S. The role of pattern-recognition receptors in innate immunity: update on Toll-like receptors. Nat Immunol. (2010) 11:373–84. 10.1038/ni.1863 20404851

[7] Jin MS Lee J-O. Structures of the toll-like receptor family and its ligand complexes. Immunity. (2008) 29:182–91. 10.1016/j.immuni.2008.07.007 18701082

[8] Takeuchi O Akira S. Pattern recognition receptors and inflammation. Cell. (2010) 140:805–20. 10.1016/j.cell.2010.01.022 20303872

[9] Medzhitov R Horng T. Transcriptional control of the inflammatory response. Nat Rev Immunol. (2009) 9:692–703. 10.1038/nri2634 19859064

[10] Takeuchi O Kawai T Muhlradt PF Morr M Radolf JD Zychlinsky A. Discrimination of bacterial lipoproteins by Toll-like receptor 6. Int Immunol. (2001) 13:933–40. 10.1093/intimm/13.7.933 11431423

[11] Alexopoulou L Holt AC Medzhitov R Flavell RA. Recognition of double-stranded RNA and activation of NF-kappaB by Toll-like receptor 3. Nature. (2001) 413:732–8. 10.1038/35099560 11607032

[12] Kagan JC Su T Horng T Chow A Akira S Medzhitov R. TRAM couples endocytosis of Toll-like receptor 4 to the induction of interferon-beta. Nat Immunol. (2008) 9:361–8. 10.1038/ni1569 18297073 PMC4112825

[13] Maurizi G Della Guardia L Maurizi A Poloni A. Adipocytes properties and crosstalk with immune system in obesity-related inflammation. J Cell Physiol. (2018) 233:88–97. 10.1002/jcp.25855 28181253

[14] Kershaw EE Flier JS. Adipose tissue as an endocrine organ. J Clin Endocrinol Metab. (2004) 89:2548–56. 10.1210/jc.2004-0395 15181022

[15] Kopp A Buechler C Neumeier M Weigert J Aslanidis C Scholmerich J. Innate immunity and adipocyte function: ligand-specific activation of multiple Toll-like receptors modulates cytokine, adipokine, and chemokine secretion in adipocytes. Obesity. (2009) 17:648–56. 10.1038/oby.2008.607 19148127

[16] Schaffler A Scholmerich J Salzberger B. Adipose tissue as an immunological organ: Toll-like receptors, C1q/TNFs and CTRPs. Trends Immunol. (2007) 28:393–9. 10.1016/j.it.2007.07.003 17681884

[17] Zhu H-J Ding H-H Deng J-Y Pan H Wang L-J Li N-S. Inhibition of preadipocyte differentiation and adipogenesis by zinc-alpha2-glycoprotein treatment in 3T3-L1 cells. J Diabetes Investig. (2013) 4:252–60. 10.1111/jdi.12046 24843663 PMC4015661

[18] Charriere G Cousin B Arnaud E Andre M Bacou F Penicaud L. Preadipocyte conversion to macrophage. Evidence of plasticity. J Biol Chem. (2003) 278:9850–5. 10.1074/jbc.M210811200 12519759

[19] Cinti S. Transdifferentiation properties of adipocytes in the adipose organ. Am J Physiol Endocrinol Metab. (2009) 297:E977–86. 10.1152/ajpendo.00183.2009 19458063

[20] Gimble J Guilak F. Adipose-derived adult stem cells: isolation, characterization, and differentiation potential. Cytotherapy. (2003) 5:362–9. 10.1080/14653240310003026 14578098

[21] Deng T Lyon CJ Minze LJ Lin J Zou J Liu JZ. Class II major histocompatibility complex plays an essential role in obesity-induced adipose inflammation. Cell Metab. (2013) 17:411–22. 10.1016/j.cmet.2013.02.009 23473035 PMC3619392

[22] Desruisseaux MS Nagajyothi Trujillo ME Tanowitz HB Scherer PE. Adipocyte, adipose tissue, and infectious disease. Infect Immun. (2007) 75:1066–78. 10.1128/IAI.01455-06 17118983 PMC1828569

[23] Blankley S Graham CM Howes A Bloom CI Berry MP Chaussabel D Identification of the key differential transcriptional responses of human whole blood following TLR2 or TLR4 ligation *in-vitro*. PLoS ONE. (2014) 9:e97702 10.1371/journal.pone.0097702 24842522 PMC4026482

[24] Lin B Dutta B Fraser ID. Systematic investigation of multi-TLR sensing identifies regulators of sustained gene activation in macrophages. Cell Syst. (2017) 5:25–37.e3. 10.1016/j.cels.2017.06.014 28750197 PMC5584636

[25] Kopp A Buechler C Bala M Neumeier M Scholmerich J Schaffler A Toll-like receptor ligands cause proinflammatory and prodiabetic activation of adipocytes via phosphorylation of extracellular signal-regilated kinase and c-jun N-terminal kinase but not interferon regulatory factor-3. Endocrinology. (2010) 151:1097–108. 10.1210/en.2009-1140 20130114

[26] Ziegler A Gonzalez L Blikslager A. Large animal models: the key to translational discovery in digestive disease research. Cell Mol Gastroenterol Hepatol. (2016) 2:716–24. 10.1016/j.jcmgh.2016.09.003 28090566 PMC5235339

[27] Sanosaka M Minashima T Suzuki K Watanabe K Ohwada S Hagino A. A combination of octanoate and oleate promotes in vitro differentiation of porcine intramuscular adipocytes. Comp Biochem Physiol B Biochem Mol Biol. (2008) 149:285–92. 10.1016/j.cbpb.2007.09.019 17977041

[28] Suzuki M Tada A Kanmani P Watanabe H Aso H Suda Y. Advanced application of porcine intramuscular adipocytes for evaluating anti-adipogenic and anti-inflammatory activities of immunobiotics. PLoS ONE. (2015) 10:e0119644. 10.1371/journal.pone.0119644 25789857 PMC4366390

[29] Mudunuri U Che A Yi M Stephens RM. bioDBnet: the biological database network. Bioinformatics. (2009) 25:555–6. 10.1093/bioinformatics/btn654 19129209 PMC2642638

[30] Huang DW Sherman BT Lempicki RA Bioinformatics enrichment tools: paths toward the comprehensive functional analysis of large gene lists. Nucleic Acids Res. (2009) 37:1–13. 10.1093/nar/gkn923 19033363 PMC2615629

[31] Xia J Benner MJ Hancock RE. NetworkAnalyst–integrative approaches for protein-protein interaction network analysis and visual exploration. Nucleic Acids Res. (2014) 42:W167–74. 10.1093/nar/gku443 24861621 PMC4086107

[32] Breuer K Foroushani AK Laird MR Chen C Sribnaia A Lo R. InnateDB: systems biology of innate immunity and beyond–recent updates and continuing curation. Nucleic Acids Res. (2013) 41:D1228–33. 10.1093/nar/gks1147 23180781 PMC3531080

[33] Wang S Sun H Ma J Zang C Wang C Wang J. Target analysis by integration of transcriptome and ChIP-seq data with BETA. Nat Protoc. (2013) 8:2502–15. 10.1038/nprot.2013.150 24263090 PMC4135175

[34] Bustin SA Benes V Garson JA Hellemans J Huggett J Kubista M. The MIQE guidelines: minimum information for publication of quantitative real-time PCR experiments. Clin Chem. (2009) 55:611–22. 10.1373/clinchem.2008.112797 19246619

[35] Nygard A-B Jorgensen CB Cirera S Fredholm M. Selection of reference genes for gene expression studies in pig tissues using SYBR green qPCR. BMC Mol Biol. (2007) 8:67. 10.1186/1471-2199-8-67 17697375 PMC2000887

[36] Ghaben AL Scherer PE. Adipogenesis and metabolic health. Nat Rev Mol Cell Biol. (2009) 20:242–58. 10.10.1038/s41580-018-0093-z 30610207

[37] Cipolletta D Feuerer M Li A Kamei N Lee J Shoelson SE. PPAR-gamma is a major driver of the accumulation and phenotype of adipose tissue Treg cells. Nature. (2012) 486:549–53. 10.1038/nature11132 22722857 PMC3387339

[38] Mo D Yu K Chen H Liu X He Z Cong P. Transcriptome landscape of porcine intramuscular adipocyte during differentiation. J. Agric. Food Chem.. (2017) 65:6317–28. 10.1021/acs.jafc.7b02039 28673084

[39] Ajuwon KM Banz W Winters TA. Stimulation with Peptidoglycan induces interleukin 6 and TLR2 expression and a concomitant downregulation of expression of adiponectin receptors 1 and 2 in 3T3-L1 adipocytes. J Inflamm. (2009) 6:8. 10.1186/1476-9255-6-8 19348674 PMC2670298

[40] Schmid A Karrasch T Thomalla M Schlegel J Salzberger B Schaffler A. Innate immunity of adipose tissue in rodent models of local and systemic staphylococcus aureus infection. Mediat Inflamm. (2017) 2017:5315602. 10.1155/2017/5315602 28428684 PMC5385907

[41] Moresco EM LaVine D Beutler B. Toll-like receptors. Curr Biol. (2011) 21: R488–93. 10.1016/j.cub.2011.05.039 21741580

[42] Uematsu S Akira S. Toll-like receptors and type I interferons. J Biol Chem. (2007) 282:15319–23. 10.1074/jbc.R700009200 17395581

[43] Uematsu S Akira S. Toll-like receptors and innate immunity. J Mol Med. (2006) 84:712–25. 10.1007/s00109-006-0084-y 16924467

[44] Meijer K de Vries M Al-Lahham S Bruinenberg M Weening D Dijkstra M. Human primary adipocytes exhibit immune cell function: adipocytes prime inflammation independent of macrophages. PLoS ONE. (2011) 6:e17154. 10.1371/journal.pone.0017154 21448265 PMC3063154

[45] Wang L Li X Wang Y. GSK3β inhibition attenuates LPS-induced IL-6 expression in porcine adipocytes. Sci. Rep. (2018) 8:15967. 10.1038/s41598-018-34186-0. 30374048 PMC6206029

[46] Vielma SA Klein RL Levingston CA Young MR. Adipocytes as immune regulatory cells. Int Immunopharmacol. (2013) 16:224–31. 10.1016/j.intimp.2013.04.002 23587489 PMC3752601

[47] Honda K Taniguchi T. IRFs: master regulators of signalling by Toll-like receptors and cytosolic pattern-recognition receptors. Nat Rev Immunol. (2006) 6:644–58. 10.1038/nri1900 16932750

[48] Ballak DB van Asseldonk EJP van Diepen JA Jansen H Hijmans A Joosten LAB TLR-3 is present in human adipocytes, but its signalling is not required for obesity-induced inflammation in adipose tissue *in vivo*. PLoS ONE. (2015) 10: e0123152 10.1371/journal.pone.0123152 25867514 PMC4395029

[49] Yu L Yan K Liu P Li N Liu Z Zhu W. Pattern recognition receptor-initiated innate antiviral response in mouse adipose cells. Immunol Cell Biol. (2014) 92:105–15. 10.1038/icb.2013.66 24165978

[50] Zhang L-J Guerrero-Juarez CF Hata T Bapat SP Ramos R Plikus MV. Innate immunity. Dermal adipocytes protect against invasive Staphylococcus aureus skin infection. Science. (2015) 347:67–71. 10.1126/science.1260972 25554785 PMC4318537

[51] Gardy JL Lynn DJ Brinkman FS Hancock RE. Enabling a systems biology approach to immunology: focus on innate immunity. Trends Immunol. (2009) 30:249–62. 10.1016/j.it.2009.03.009 19428301

[52] Zhang X Kimura Y Fang C Zhou L Sfyroera G Lambris JD. Regulation of Toll-like receptor-mediated inflammatory response by complement *in vivo*. Blood. (2007) 110:228–36. 10.1182/blood-2006-12-063636 17363730 PMC1896115

[53] Moue M Tohno M Shimazu T Kido T Aso H Saito T. Toll-like receptor 4 and cytokine expression involved in functional immune response in an originally established porcine intestinal epitheliocyte cell line. Biochim Biophys Acta. (2008) 1780:134–44. 10.1016/j.bbagen.2007.11.006 18082146

[54] Roca-Rivada A Bravo SB Pérez-Sotelo D Alonso J Castro AI Baamonde I. CILAIR-based secretome analysis of obese visceral and subcutaneous adipose tissues reveals distinctive ECM remodeling and inflammation mediators. Sci. Rep. (2015) 5:12214. 10.1038/srep12214. 26198096 PMC4648467

[55] Gardan D Gondret F Louveau I. Lipid metabolism and secretory function of porcine intramuscular adipocytes compared with subcutaneous and perirenal adipocytes. Am J Physiol Endocrine Metab. (2006) 291:E372–80. 10.1152/ajpendo.00482.2005 16705057

[56] Rosen ED Spiegelman BM. Adipocytes as regulators of energy balance and glucose homeostasis. Nature. (2006) 444:847–53. 10.1038/nature05483 17167472 PMC3212857

[57] Hegde V Dhurandhar NV. Microbes and obesity-interrelationship between infection, adipose tissue and the immune system. Clin Microbiol Infect. (2013) 19:314–20. 10.1111/1469-0691.1215 23506525

[58] Wang B Fu X Liang X Deavila JM Wang Z Zhao L. Retinoic acid induces white adipose tissue browning by increasing adipose vascularity and inducing beige adipogenesis of PDGFRalpha+ adipose progenitors. Cell Discov. (2017) 3:17036. 10.1038/celldisc.2017.36 29021914 PMC5633810

